# The Trypanocidal Activity of Naphthoquinones: A Review

**DOI:** 10.3390/molecules14114570

**Published:** 2009-11-10

**Authors:** Antônio Ventura Pinto, Solange Lisboa de Castro

**Affiliations:** 1 Núcleo de Pesquisas em Produtos Naturais, Centro de Ciências da Saúde, Universidade Federal do Rio de Janeiro, 21944-970, Rio de Janeiro, RJ, Brazil; 2 Laboratório de Biologia Celular, Instituto Oswaldo Cruz, Fundação Oswaldo Cruz, 21045-900, Rio de Janeiro, RJ, Brazil

**Keywords:** naphthoquinones, β-lapachone, chagas disease, *Trypanosoma cruzi*

## Abstract

Naphthoquinones are compounds present in several families of higher plants. Their molecular structures confer redox properties, and they are involved in multiple biological oxidative processes. In folk medicine, especially among Indian populations, plants containing naphthoquinones have been employed for the treatment of various diseases. The biological redox cycle of quinones can be initiated by one electron reduction leading to the formation of semiquinones, unstable intermediates that react rapidly with molecular oxygen, generating free radicals. Alternatively, the reduction by two electrons, mediated by DT-diphorase, leads to the formation of hydroquinone. Lapachol, α-lapachone and β-lapachone, which are isolated from the heartwood of trees of the Bignoniaceae family, are examples of bioactive naphthoquinones. In this review, we will discuss studies investigating the activity of these natural products and their derivatives in the context of the search for alternative drugs for Chagas disease, caused by *Trypanosoma cruzi*, a neglected illness that is endemic in Latin America.

## Introduction

For thousands of years, medicine and natural products have been closely linked through the use of traditional medicines and natural poisons [1]. China and India have a well-established herbal medicine industries, providing interesting new drug leads for potential development in Western medicine. In France and Germany, medical herbalism continues to co-exist with modern pharmacology, albeit at an increasingly lower level [2]. However, the benefits of modern drugs are felt primarily in developed countries, while developing countries continue to rely on ethnobotanical remedies as their primary medicines, leaving approximately 80% of the world’s population without access to modern healthcare products [3,4,5].

Natural products are typically secondary metabolites, produced by plants, microorganisms and animals in response to external stimuli such as nutritional changes. They are widely recognised in the pharmaceutical industry for their remarkable structural diversity and wide range of pharmacological activities. Pharmacophores derived from natural products are well represented in lists of “privileged structures”, which makes them excellent candidates for building blocks for biologically relevant chemical libraries [6].

Inspection of the rate of NCE (New Chemical Entities) approvals demonstrates that the natural products field was still producing or was involved in about 50% of all small molecules during the years 2000-2006, and that during this time, a significant number of NCEs (83 of 264) were biologicals or vaccines [7]. The impact of natural products on drug discovery will remain considerable for many years to come, not only for cancer, but also for diseases such as microbial and parasitic infections [8]. An increasing awareness of the potential of natural products may lead to the development of much-needed new drugs for parasitic diseases [9], which remain a major public health problem, particularly in tropical developing countries. The limited availability and affordability of pharmaceutical medicines means that the majority of the world's population depends on traditional medical remedies. An increased understanding of the modes of action of plant medicinal extracts, together with the availability of parasite genome sequences, has intensified the search for novel antiparasitic drugs.

## Napththoquinones as Privileged Molecules

Naphthoquinones are considered privileged structures in medicinal chemistry due to their biological activities and structural properties [10]. They are present in various families of plants and serve as vital links in the electron transport chains in the metabolic pathway, participating in multiple biological oxidative processes [11,12]. The fundamental feature of quinone chemistry is its ease of reduction and, therefore, its ability to act as an oxidising or dehydrogenating agent. This redox property is driven by the formation of a fully aromatic system [13,14]. In folk medicine, plants containing naphthoquinones are often employed for the treatment of various diseases [15,16], and several quinonoids isolated from traditional medicinal plants are being investigated for their anticancer properties [17]. 

**Scheme 1:**
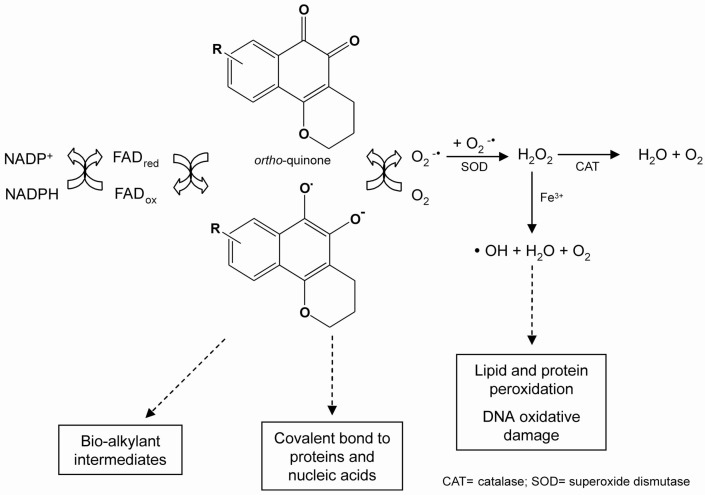
Representation of the redox cycle and generation of metabolites by quinones.

The redox cycling of quinones may be initiated by either a one- or two-electron reduction. The one-electron reduction of quinones is catalysed by NADPH-cytochrome P450 reductase, and yields unstable semiquinones. Quinones transfer electrons to molecular oxygen (O_2_), and return to their original quinoidal formation, thus generating a superoxide anion radical (^.^O_2_^-^). Superoxide can be converted to hydrogen peroxide (H_2_O_2_) via a superoxide dismutase (SOD)-catalysed reaction, followed by the formation of a hydroxyl radical (^.^HO) by the iron-catalysed reduction of peroxide via the Fenton reaction. All of these highly reactive species may react directly with DNA or other cellular macromolecules, such as lipids and proteins, leading to cell damage [18]. This reaction results in the shunting of electrons toward oxygen, a futile pathway for reduction equivalents otherwise used for cytochrome P450 reductase-dependent reactions (Scheme 1).

The two-electron reduction of quinones is catalysed by NAD(P)H:[quinone acceptor]oxidoreductase (NQO1, DT-diaphorase, EC 1.6.99.2) [19], and generates hydroquinones (QH_2_). This enzyme reduces toxic, reactive and unstable quinones, bypassing the creation of toxic intermediates (e.g. a semiquinone radical), and sparing the cell from ROS formation. Whether the two-electron reduction of a quinone leads to detoxiﬁcation or to activation of oxidative stress depends upon the rate of autooxidation of the formed hydroquinone [20]. If this rate is low under physiological conditions, conjugation may occur before oxidation. As a consequence, the two-electron reduction will lead to detoxiﬁcation, and an increase in the NQO1 activity in tissues would be expected to decrease the toxicity of the quinone. If, however, the hydroquinone is rapidly oxidised, only a minor fraction may be conjugated before oxidation occurs, and hydroquinone formation would constitute an activation reaction. As a result, enhanced tissue levels of NQO1 would be expected to increase the toxicity of the quinone [21].

Quinones are oxidants and electrophiles, and the relative contribution of these properties to both their toxic and therapeutic activities is influenced by their chemical structure, particularly substituent effects and the characteristics of the quinone nucleus [22]. Two major mechanisms of quinone cytotoxicity have been proposed: stimulation of oxidative stress and alkylation of cellular nucleophiles, which encompass a large range of biomolecules [23]. ROS may react directly with DNA, lipids and proteins, leading to cell damage [24,25,26,27] and shunting electrons toward oxygen, a futile pathway for reduction equivalents otherwise used for cytochrome P450 reductase-dependent reactions. Cellular damage can also occur through the alkylation of crucial proteins and nucleic acids. 

## Biological Activity of β-Lapachone

**Figure 1:**
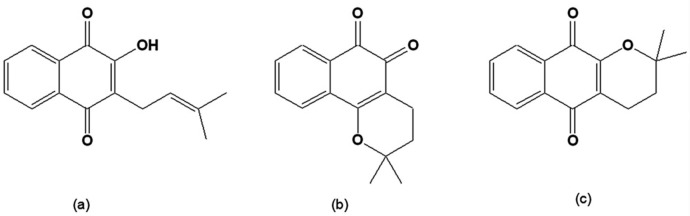
(a) 2-Hydroxy-3-(3´-methyl-2-butenyl)-1,4-naphthoquinone [lapachol]; (b) 2,2-Dimethyl-3,4-dihydro-2*H*-benzo[*h*]chromene-5,6-dione [β-lapachone]; (c) 2,2-Dimethyl-3,4-dihydro-2H-benzo[g]chromene-5,10-dione [α-lapachone].

The bioactive quinones include lapachol, α-lapachone and β-lapachone (Figure 1), originally isolated from the heartwood of trees of the Bignoniaceae family (*Tabebuia sp*). They can also be found in other families such as Verbenaceae, Proteaceae, Leguminosae, Sapotaceae, Scrophulariaceae, and Malvaceae [28]. The inner bark of *Tabebuia avellanedae*, commonly known as "pau d'arco" (lapacho, taheebo), is used as an analgesic, an anti-inflammatory, an antineoplasic and a diuretic by the local people in the northeastern regions of Brazil [29]. The synthesis and chemistry of β-lapachone and related compounds was initially investigated in the beginning of the twentieth century by the chemist Samuel Hooker [28]. 

β-Lapachone was found to be cytotoxic to a variety of human cancers [29,30,31,32,33]. This naphthoquinone is now under investigation for the treatment of specific cancers associated with elevated NQO1 levels, such as breast, non-small cell lung, pancreatic, colon, and prostate cancers [34,35,36,37], and is currently in phase II clinical trials for the treatment of pancreatic cancer [38]. Particularly promising is the synergistic lethality of β-lapachone with taxol [39] and genistein [40] on several tumour cell lines implanted into mice. DNA topoisomerase I was the first biochemical target of β-lapachone to be reported. β-Lapachone acts on this enzyme in a manner distinct from that of other known inhibitors, such as campthotecin [41,42]. Topoisomerases are essential enzymes in the regulation of DNA topology; type I catalyses the relaxation of positive supercoiling by single strand breaks, while type II makes a transient double-strand break [43]. Subsequently, β-lapachone was reported as a weak topoisomerase II poison, a reaction that is independent of ATP and involves the formation of reversible cleavable complexes [44]. It was also hypothesised that the cytotoxic actions of naphthoquinones are derived in part from the alkylation of exposed thiol residues on topoisomerase II–DNA complexes [45]. β-Lapachone inhibited the enzyme by inducing its religation and dissociation from DNA in the presence of ATP [46]. The diverse and unique mechanisms of topoisomerase II inhibition by naphthoquinone derivatives reveal novel ways to target the enzyme with the potential for anti-cancer drug design. 

β-Lapachone induces a novel caspase- and p53-independent cell death pathway in human cancer cell lineages overexpressing NQO1. This enzyme substantially enhances the toxicity of β-lapachone by reducing the quinone to an unstable hydroquinone, which rapidly undergoes a two-step oxidation back to the parent compound, perpetuating a futile redox cycle [34,47]. Either apoptotic or necrotic cell death can result, as reported in various studies performed under various conditions [39,48].

## Chagas Disease, a Neglected Disease

The neglected tropical diseases are a group of infections caused by worms, bacteria and pathogenic trypanosomatids (Chagas disease, African trypanosomiasis and leishmaniasis) that affect at least 2.7 billion people worldwide [49]. However, less than 1% of the new drugs introduced into our therapeutic arsenal over the last 30 years have been directed to tropical diseases [50,51,52,53]. The cost of investments and the lack of potential and security markets in developing countries have dampened interest in developing drugs for tropical diseases. Chagas disease, caused by the protozoan *Trypanosoma cruzi*, is responsible for considerable human mortality and morbidity [53]. Although it was first described one hundred years ago by Carlos Chagas [54], this disease still represents an important health problem in Latin America [55]. The life cycle of *T. cruzi* involves a hematophagous triatomine insect, a vertebrate host and different forms of the parasite. Briefly, a bloodstream trypomastigote ingested by the insect differentiates into an epimastigote, which proliferates and, in the posterior intestine, differentiates into the metacyclic form. This infective form invades the vertebrate cell and undergoes differentiation into the intracellular amastigote, which proliferates and then transforms into the trypomastigote, the form that disseminates the infection. 

In humans, during the acute phase of Chagas disease and in the absence of specific treatment, the symptoms persist for about two months, with a mortality of 2 to 8%, especially among children. In the chronic phase, most patients remain asymptomatic, but about 20% of cases develop the symptoms characteristic of this phase, namely cardiac, digestive or neurologic disturbances [56]. Thus, Chagas is a major cause of infectious cardiac disease in endemic areas [57]. The transmission of this disease occurs primarily via the vector, with blood transfusion and congenital transmission being somewhat less common [58]. Instances of transmission via laboratory accident [59], organ transplantation [60,61] and ingestion of infected food or contaminated insects [62,63] have also been reported. Recently, Chagas disease has also been recognised as an opportunistic disease in HIV-infected individuals [64]. In addition, it is now being reported throughout the world due to international immigration [65].

**Figure 2:**
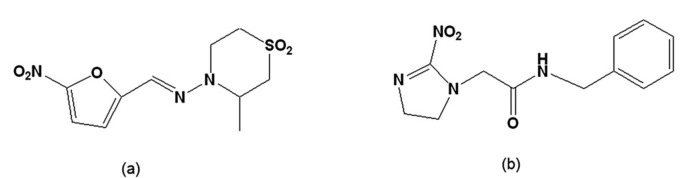
(a) Tetrahydro-3-methyl-4-[(5-nitrofurfurylidene)amino-2*H*-1,4-thiazine 1,1,-dioxide [nifurtimox]; (b) N-benzyl-2-nitroimidazole acetamide [benznidazole].

Although the occurrence of acute cases has sharply declined due to Southern Cone Initiative efforts toward vector transmission control [66], we still face considerable challenges, including the maintenance of sustainable public policies regarding Chagas disease control and the urgent need for better drugs to treat chagasic patients. Introduced in the 1960s and 1970s, the nitroderivatives nifurtimox and benznidazole (Figure 2) were the most commonly used drugs for treatment of this disease. While these are effective for acute infections, the data regarding their use and efficacy during the chronic phase are still controversial. This controversy is primarily due to the undesirable side effects that frequently force the abandonment of treatment, poor indices of apparent cure and a lack consensus about the available criteria for the evaluation of parasitological cure during this later phase of the disease [67]. As is also the case for other neglected diseases, drugs for Chagas disease are not of interest to pharmaceutical industries. After the introduction of nifurtimox and benznidazole, despite the extensive list of classes of compounds with *in vitro* and *in vivo* activity against *T. cruzi*, with the exception of allopurinol, itraconazole and fluconazole, none was submitted to clinical trials [68].

## Trypanocidal Activity of β-Lapachone and Naphthoquinone Derivatives

Besides their widespread presence in nature, the great interest in the study and mechanisms of action of compounds with a quinoidal structure is due to their multiple roles in organisms. The antiprotozoal activities of naphthoquinones have been reported, and several of them have been identiﬁed as possible leads for drug development [69,70,71,72].

The effect of lapachol, β-lapachone and their derivatives on *T. cruzi* was investigated by Drs. Stoppani, Cruz and DoCampo in Argentina and Brazil. The addition of β-lapachone to intact *T. cruzi* epimastigotes, or to mitochondrial or microsomal fractions, together with NADH or NADPH, induced the release of superoxide anion radical and H_2_O_2_. Ultrastructural analysis of treated amastigotes and trypomastigotes revealed the rearrangment of the chromatin into patches, alterations of the nuclear and cytoplasmic membranes and mitochondrial swelling [73,74,75]. β-Lapachone also inhibited DNA, RNA and, to a lesser extent, protein synthesis [76]. Unfortunately, no trypanocidal effect was observed in suspensions containing foetal calf serum or rabbit haemoglobin solution, suggesting that β-lapachone could be inactivated either by reduction in the presence of oxyhaemoglobin or by interaction with serum proteins [77]. Such deactivation could occur through reaction of the quinone with proteins present in the blood, specifically via interaction of the quinoidal moiety with free basic NH_2_ residues of proteins. The synthetic analogue allyl-β-lapachone was not inactivated in the presence of blood and remained effective in suppressing trypomastigote infectivity, and is considered a potential chemoprophylactic agent for use in blood banks [78]. Like β-lapachone, this derivative inhibited epimastigote proliferation, leading to alterations in cellular membranes, chromatin structure and the mitochondrion, and an increase in respiratory rate, H_2_O_2 _generation and lipid peroxidation [77,79].

**Figure 3:**
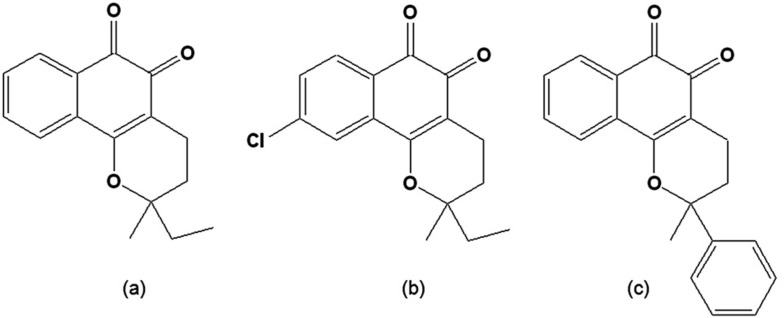
(a) 3,4-Dihydro-2-methyl-2-ethyl-2*H*-naphtho[1,2*b*]pyran-5,6-dione [CG8-935]; (b) 3,4-Dihydro-2-methyl-2-phenyl-2*H*-naphtho[1,2b]pyran-5,6-dione [CG9-442] (c) 3,4-Dihydro-2,2-dimethyl-9-chloro-2*H*-naphtho[1,2*b*]pyran-5,6-dione [CG10-248].

A series of *o*-naphthoquinones named CG8-935 (3,4-dihydro-2-methyl-2-ethyl-2H-naphtho[1,2*b*]-pyran-5,6-dione), CG9-442 (3,4-dihydro-2-methyl-2-phenyl-2H-naphtho[1,2*b*]pyran-5,6-dione, 2-phenyl-β-lapachone) and CG10-248 (3,4-dihydro-2,2-dimethyl-9-chloro-2H-naphtho[1,2*b*]pyran-5,6-dione) (Figure 3) were investigated by Stoppani and colleagues [80,81]. Of these compounds, CG9-442 proved to be the most active in inducing oxidative damage in trypanosomatids [82]. The contribution of oxygen radical production to quinone cytotoxicity was supported by the spectroscopic observation of β-lapachone, CG 8-935, CG 9-442 and CG 10-248 redox cycling, as well as by the production of the semiquinone radical, superoxide anion radical and H_2_O_2_ and the effect of this naphthoquinones on cell respiration. In the same experimental conditions, the *p*-naphthoquinones, α-lapachone and menadione, were barely active [82].

**Figure 4:**
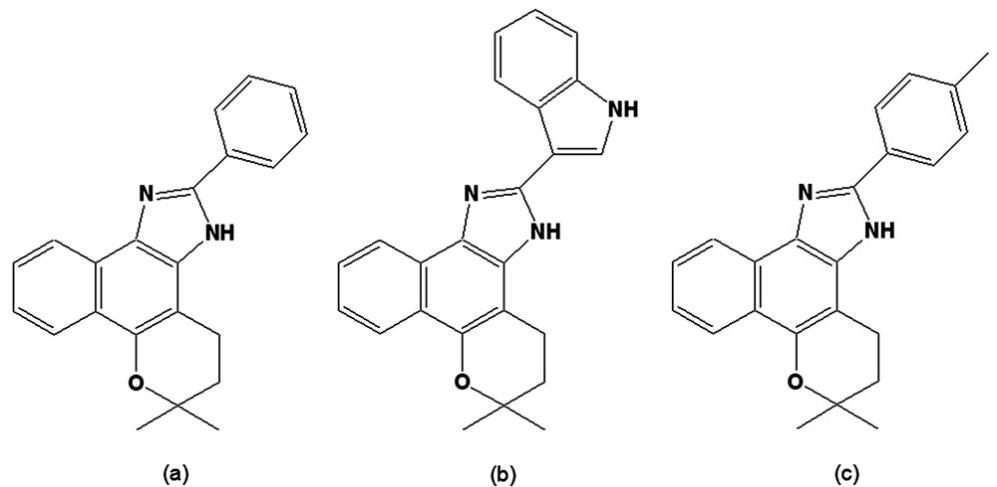
(a) 4,5-Dihydro-6,6-dimethyl-6*H*-2-(phenyl)-pyran[*b*-4,3]naphth[1,2-d]imidazole [N1]; (b) 4,5-Dihydro-6,6-dimethyl-6*H*-2-(3′-indolyl)-pyran[*b*-4,3]naphth[1,2-d]imidazole] [N2]; (c) 4,5-Dihydro-6,6-dimethyl-6*H*-2-(4′-methylphenyl)-pyran[*b*-4,3]naphth[1,2-d]-imidazole [N3].

Due to the easy access to natural sources of quinones from Brazilian flora and the facile synthetic routes previously developed by one of the authors (AVP) when exploring the reactivity of 1,2-quinoidal carbonyls [83,84,85,86,87], naphthoquinones were taken as starting points for screening trypanocidal drugs. Fifty-four derivatives were obtained through the reaction of naphthoquinones with common reagents from heterocyclic chemistry, leading to the synthesis of 14 oxazoles, 30 imidazoles and 10 other related heterocyclic compounds [88,89,90,91]. The evaluation of these compounds, together with the original natural naphthoquinones, was performed using bloodstream trypomastigote forms of *T. cruzi*,as previously described [92]. By comparing the activities of the original naphthoquinones to the synthetic compounds, we concluded that structural features involved in increases in lipophilicity, such as a furan moiety or the presence of a methoxyl group or an aliphatic side chain, led to an increase in the trypanocidal activity. It is possible that an increase in lipophilic character allows for better penetration of the compound through the plasma membrane of the parasite. For the naphthooxazoles assayed, there was no clear correlation between biological activity and the type of the mono-oxygenated ring (pyran *versus* furan). As was the case for the naphthoquinones, the presence of a methoxy group, phenyl group or other lipophilic groups increased the trypanocidal activity. Most of the synthesised naphthoimidazoles contained phenyl units with either electron-releasing or electron-withdrawing groups attached to the imidazole ring, and the results obtained suggested that electronic factors were not important for the biological effect [93,94]. Among all the investigated compounds, three naphthoimidazoles derived from β-lapachone, with the aromatic moieties linked to the imidazole ring N1 (4,5-dihydro-6,6-dimethyl-6*H*-2-(phenyl)-pyran[b-4,3]naphth[1,2-d]imidazole), N2 (4,5-dihydro-6,6-dimethyl-6*H*-2-(3′-indolyl)-pyran[*b*-4,3]naphth[1,2-d] imidazole) and N3 (4,5-dihydro-6,6-dimethyl-6*H*-2-(4′-methylphenyl)-pyran[*b*-4,3]naphth[1,2-d]imidazole) (Figure 4) showed the highest activity on trypomastigote forms and were further investigated. These molecules were also active on intracellular amastigotes and epimastigotes, and presented low toxicity to host cells. In epimastigotes, the compounds blocked the cell cycle and metacyclogenesis, inhibited succinate cytochrome c reductase and induced damage to the mitochondrion, Golgi complex and reservosomes. In trypomastigotes, the compounds caused alterations in the kinetoplast and mitochondrion, plasma membrane blebbing and DNA fragmentation [95,96]. The mitochondrion, reservosomes and DNA were identified as their main targets in *T. cruzi*. The strong increase in labelling of monodansyl cadaverine, the inhibition of the death process by wortmannin or 3-methyladenine and the overexpression of ATG genes in treated parasites, together with ultrastructural evidence, point to autophagy as the predominant phenotype induced by the naphthoimidazoles [97,98]. Their precise mode of action upon the parasite appears to be complex; however, we can exclude damage caused by oxidative stress since, unlike the original napthoquinones, N1, N2 and N3 do not easily undergo redox reactions. It is important to note that several trypanocidal agents, such as benznidazole, contain imidazole moieties [99,100,101,102], which is consistent with the idea that the trypanocidal activity is associated with the imidazole skeleton.

**Figure 5:**
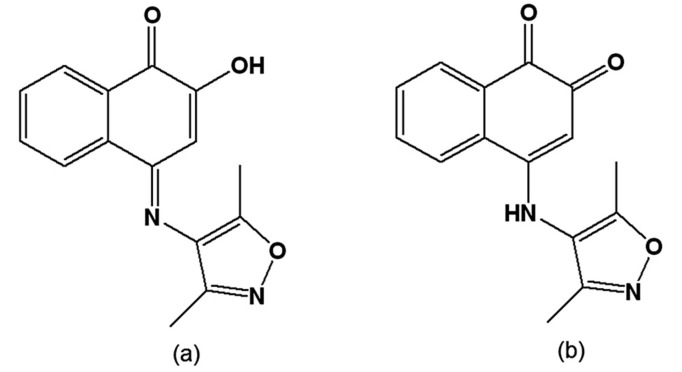
(a) (*E*)-4-(3,5-Dimehtylisoxazol-4-ylimino)-2-hydroxynapththalene-1(4*H*)-one; (b) 4-(3,5-Dimethylisoxazol-4-ylamino)naphthalene-1,2-dione.

A series of synthetic isoxazolylnaphthoquinones was assayed *in vitro* and *in vivo* on *T. cruzi*, and the most active was (*E*)-4-(3,5-dimethylisoxazol-4-ylimino)-2-hydroxynapththalene-1(4*H*)-one (Figure 5a), which led to parasitemia reduction in infected mice [103]. In the epimastigote, this compound inhibited growth and DNA synthesis and stimulated O_2_ uptake and superoxide anion radical (O_2_^-.^) generation by the parasite and by its mitochondrial and microsomal membranes, indicating the involvement of free radicals in its activity [104]. More recently, the *in vitro* trypanocidal effect of 4-(3,5-dimethylisoxazol-4-ylamino)naphthalene-1,2-dione (Figure 5b) was reported [105], but the compound was also toxic to murine L-6 cells. In an attempt to circumvent this issue, new derivatives were synthesised; however, while these displayed trypanocidal effects, they were also toxic to mammalian cells [106].

**Figure 6:**
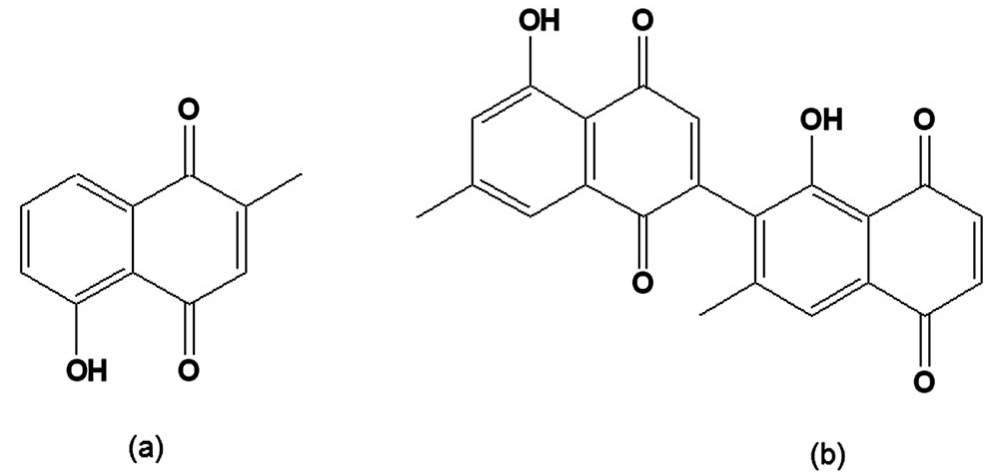
(a) 5-Hydroxy-2-methyl-1,4-naphthoquinone [plumbagin]; (b) [1´,5-Dihydroxy-3´,7-dimethyl-2,2´-binaphthalene-1,4,5´,8´-tetrone 2,6´-bis(5-hydroxy-7-methyl-1,4-naphthoquinone [diospyrin].

Plumbagin (Figure 6a) has been found to be active on epimastigote forms, leading to the total lysis of bloodstream trypomastigotes at a concentration similar to that of crystal violet, the standard drug recommended for the chemoprophylaxis of banked blood [107,108]. The related diospyrin (Figure 6b), a dimer of 7-methyljuglone isolated from the Indian plant *Diospyros montana* [109] and four synthetic derivatives were assayed on intracellular forms of *T. cruzi*. The dimethyl derivative was found to be more active than the parent compound [110].

**Figure 7:**
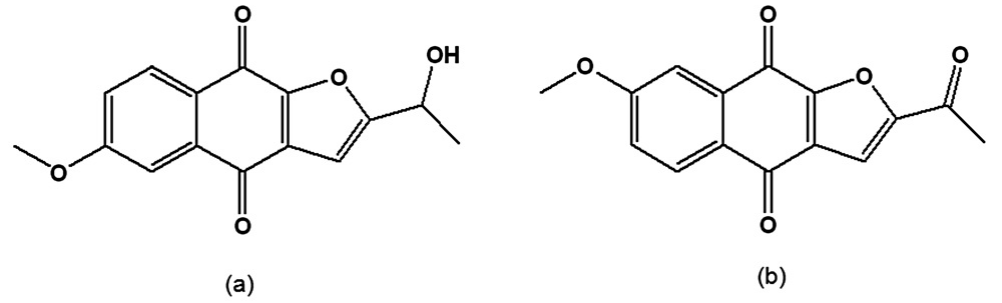
(a) 2-(1-Hydroxyethyl)-6-methoxy-naphtho[2,3-*b*]furan-4,9-quinone; (b) 2-Acetyl-7-methoxy-naphtho[2,3-*b*]furan-4,9-quinone.

When synthetic and natural naphthofuranquinones were assayed on epimastigotes of *T. cruzi* strains with different susceptibilities to benznidazole, the most active were 2-(1-hydroxyethyl)-6-methoxy-naphtho[2,3-*b*]furan-4,9-quinone and 2-acetyl-7-methoxy-naphtho[2,3-*b*]furan-4,9-quinone (Figure 7a,b), which contain one methoxy group. However, their trypanocidal activity was independent of the parasite subpopulation [111].

**Figure 8:**
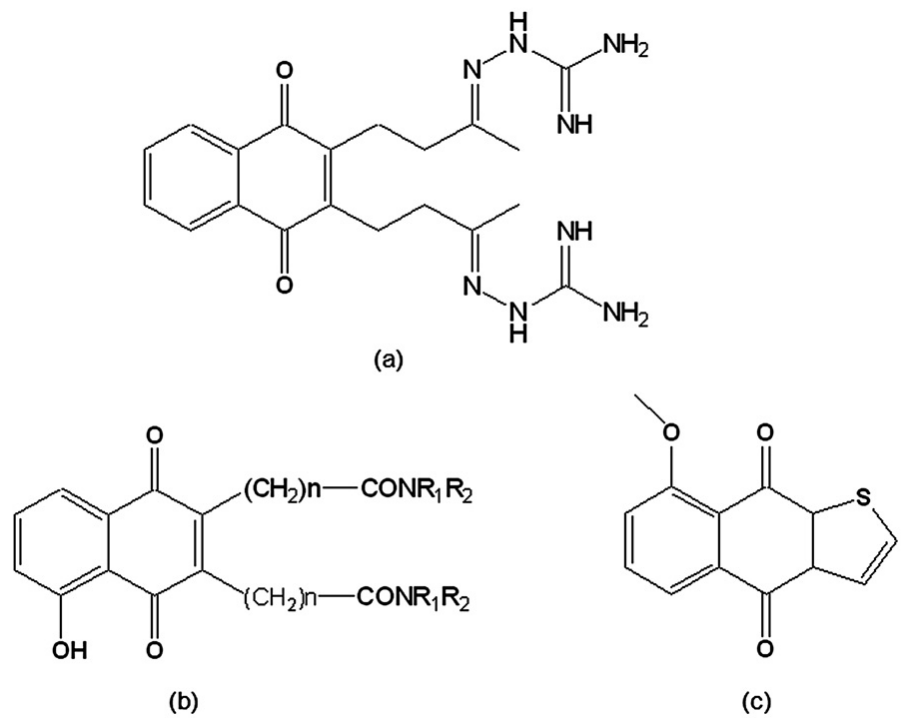
(a) 2,3-*bis*-[3-(2-Amidinohydrazono)-butyl]-1,4-naphthoquinone dihydro-chloride; (b) 3,3'-[Polyaminobis(carbonylalkyl)]-*bis*(1,4-naphthoquinones); (c) 8-Methoxy-naphtho[2,3-*b*]thiophen-4,9-quinone.

*T. cruzi*, as well as other trypanosomatids, possesses a unique thiol metabolism based on trypanothione, which is kept reduced by the parasite-specific flavoenzyme trypanothione reductase (TR), a recognised target for the chemotherapy of Chagas disease [112]. Although TR has 40% homology with the analogous human glutathione reductase, their active sites are sufficiently different to allow the development of selective TR inhibitors. In the presence of oxygen, these inhibitors are cyclically reduced and reoxidised, generating deleterious oxygen radicals and inhibiting the ability of the enzyme to reduce trypanothione disulfide, its physiological substrate. The first group of TR inhibitors reported were naphthoquinones and nitrofuranes, named “subversive substrates” [113] due to the futile-cycling of the enzyme induced by such drugs. Among these was 2,3-*bis*[3-(2-amidinohydrazono)-butyl]-1,4-naphthoquinone dihydrochloride (Figure 8a), which was active on the trypomastigote form, reducing its ability to infect host cells [113] and strongly inhibiting TR [114]. Three series of 1,4-naphthoquinones were synthesised by functionalisation at carbons 2 and/or 3 of menadione, plumbagin, and juglone by polyamine chains. When these were assayed as potential TR inhibitors, the most active compounds were from the 3,3'-[polyaminobis(carbonylalkyl)]-bis(1,4-naphthoquinones) series (Figure 8b), which exhibited potent *in vitro* activity on *T. cruzi* epimastigotes [115,116]. Naphtho[2,3-*b*]thiophen-4,9-quinone and its derivatives were also prepared and evaluated on epimastigotes and trypomastigotes as TR inhibitors. It was observed that side chains that are positively charged in physiological medium or substituents that change the redox potential of the quinoid ring were important factors in a lead compound [117]. 8-Methoxy-naphtho[2,3-*b*]thiophen-4,9-quinone (Figure 8c) was able to inhibit enzyme activity by 87% at a concentration of 100 µM after a 30 min incubation. This compound was also one of the most active among 150 natural and synthetic compounds evaluated on TR. Its mode of inhibition fits a non-competitive model with respect to the substrate (trypanothione) and to the co-factor (NADPH). In addition, when tested on human glutathione reductase, this compound did not display any significant inhibition, indicating a good selectivity for the parasite enzyme [118].

**Figure 9:**
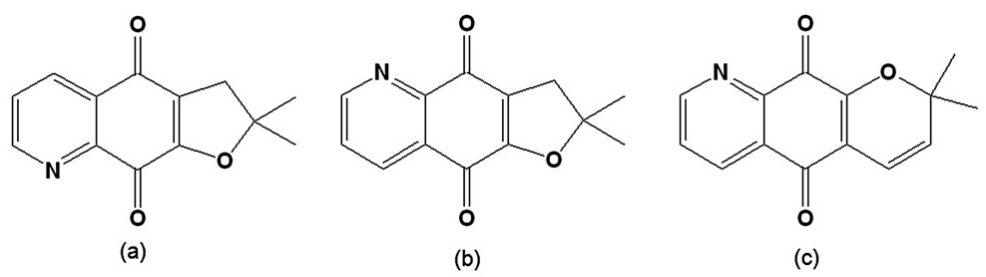
(a) 2,3-Dihydro-2,2-dimethylfuro[3,2-*g*]quinoline-4,9-dione; (b) 2,3-Dihydro-2,2-dimethylfuro[2,3-*g*]quinoline-4,9-dione; (c) 2,2-Dimethyl-2H-pyran[2,3-*g*]quinoline-5,10-dione.

A series of derivatives from 2,3-dihydrobenzo[*b*]furan-4,7-dione were prepared and assayed on *T. cruzi* epimastigotes. Two furoquinolinediones, 2,3-dihydro-2,2-dimethylfuro[3,2-*g*]quinoline-4,9-dione and 2,3-dihydro-2,2-dimethylfuro[2,3-*g*]quinoline-4,9-dione (Figure 9), were effective inhibitors of parasite proliferation, indicating that the presence of a pyridine instead of a benzene ring increases trypanocidal activity. However, only the second derivative, the 1,5-regioisomer (Figure 9b), was active as a redox cycling agent, increasing oxygen uptake. 

Analysis of the stereoelectronic properties using the density-functional theory method showed that this activity is related to the capacity of the system to acquire electronic charge from its surroundings [119]. When the activity of a series of α- and β-lapachones on epimastigotes and trypomastigotes was evaluated, it was found that the most active compound was an α-lapachone derivative with a pyridine moiety instead of a benzene ring (2,2-dimethyl-2H-pyran[2,3-*g*]quinoline-5,10-dione) (Figure 9c) [120]. This structural factor may be important for the design of new derivatives, as adding a nitrogen isosteric modiﬁcation on the aromatic ring could improve the trypanocidal activity. As noted by the authors, this is an interesting result because α-lapachones normally have a weaker trypanocidal activity [77,78]. It was previously shown via the measurement of the redox potential of a series of naphthoquinones that the ease of reduction is associated with the activity on *T. cruzi*, as angular naphthofurandiones (*o*-quinones) are more active than the linear isomers (*p*-quinones) [121].

**Figure 10:**
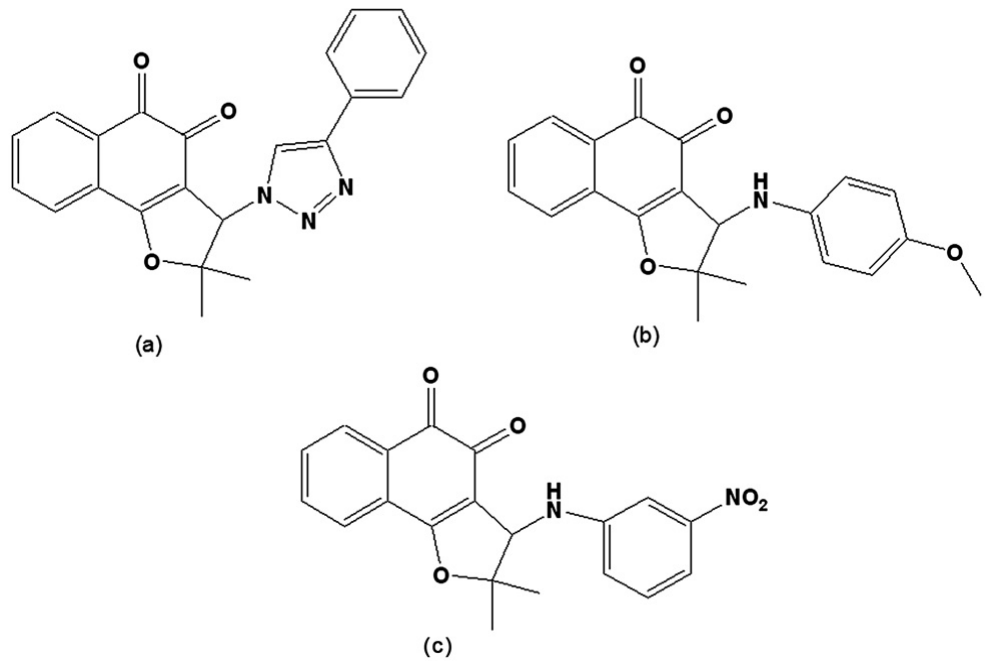
(a) 2,2-Dimethyl-3-(4-phenyl-[1,2,3]triazol-1-yl)-2,3-dihydro-naphtho[1,2-*b*]furan-4,5-dione; (b) 3-(4-methoxyphenylamino)-2,3-dihydro-2,2-dimethylnaphtho[1,2-*b*]furan-4,5-dione; (c) 3-(3-nitrophenylamino)-2,3-dihydro-2,2-dimethylnaphtho[1,2-*b*]furan-4,5-dione.

New compounds were prepared via the hybridisation of naphthoquinones and [1,2,3]-triazoles or arylamines and evaluated on trypomastigote forms. From nor-lapachol (2-hydroxy-3-(2´-methyl-1-propenyl)-1,4-naphthoquinone), several derivatives were prepared. The most active was 2,2-dimethyl-3-(4-phenyl-[1,2,3]triazol-1-yl)-2,3-dihydro-naphtho[1,2-*b*]furan-4,5-dione (Figure 10a) with a phenyl group attached to the triazolic ring, which, due to its higher lipophilic character when compared to other triazoles, allows for better penetration through the parasite’s plasma membrane [122]. From the reaction of nor-lapachol with aryl amines, 10 substituted *ortho*-naphthofuranquinones and a non-substituted *para*-naphthofuranquinone were created. The most active compounds were the *o*- naphthofuranquinones 3-(4-methoxyphenylamino)-2,3-dihydro-2,2-dimethylnaphtho[1,2-*b*]furan-4,5-dione and 3-(3-nitrophenylamino)-2,3-dihydro-2,2-dimethylnaphtho[1,2-*b*]furan-4,5-dione (Figure 10b,c), which had trypanocidal activities higher than that of benznidazole, the standard drug [123]. Such hybrid molecules, obtained from quinones and triazoles or arylamino groups, endowed the quinones with redox properties, representing an interesting starting point for a medicinal chemistry program directed toward the chemotherapy of Chagas’ disease.

**Figure 11:**
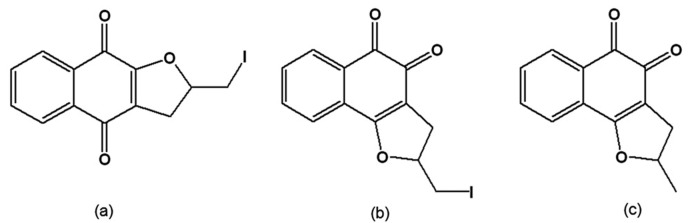
(a) 2,3-Dihydro-2-iodomethylene-4,9-dioxonaphtho[2,3-*b*]furan; (b) 2,3-Dihydro-2-iodomethylene-4,5-dioxo-naphtho[1,2-*b*]furan; (c) 2,3-Dihydro-2-methyl-4,5-dioxo-naphtho[1,2-*b*]furan.

From the reaction of C-allyl lawsone (2-hydroxy-3-allyl-1,4-naphthoquinone) with metallic iodine, the naphthofuranquinones 2,3-dihydro-2-iodomethylene-4,9-dioxonaphtho[2,3-*b*]furan (Figure 11a) and 2,3-dihydro-2-iodomethylene-4,5-dioxo-naphtho[1,2-*b*]furan (Figure 11b) were synthesised by the electrophilic addition of iodine to the allylic double bond and subsequent cyclisation, generating a furan ring. Dissolution of C-allyl lawsone in sulphuric acid led to the formation of 2,3-dihydro-2-methyl-4,5-dioxo-naphtho[1,2-*b*]furan (Figure 11c). These three quinones were active on the trypomastigote, intracellular amastigote and epimastigote forms, and had low toxicity to the host mammalian cells [124]. Ultrastructural analysis of treated epimastigotes and trypomastigotes indicated a potent effect of the compounds on the mitochondria, which were significantly swollen and possessed a washed-out matrix profile. Fluorescence-activated cell sorting analysis of rhodamine 123-stained *T. cruzi* showed that the quinones caused a dose-dependent collapse of the mitochondrial membrane potential, especially for epimastigote forms. These compounds also specifically decreased mitochondrial complex I-III activities in both epimastigotes and trypomastigotes, which paralleled the reduction in succinate-induced oxygen consumption. Mitochondrial hydrogen peroxide formation was also increased in treated epimastigotes. These results indicate that the trypanocidal action of the naphthofuranquinones is associated with mitochondrial dysfunction, which leads to increased reactive oxygen species generation and parasite death [125]. The facile synthesis of these compounds opens the possibility of large-scale production with high yields that can be assayed in experimental mouse models.
